# Physical activity from adolescence to young adulthood: patterns of change, and their associations with activity domains and sedentary time

**DOI:** 10.1186/s12966-021-01130-x

**Published:** 2021-06-30

**Authors:** Tuula Aira, Tommi Vasankari, Olli Juhani Heinonen, Raija Korpelainen, Jimi Kotkajuuri, Jari Parkkari, Kai Savonen, Arja Uusitalo, Maarit Valtonen, Jari Villberg, Henri Vähä-Ypyä, Sami Petteri Kokko

**Affiliations:** 1 Faculty of Sport and Health Sciences, Research Centre for Health Promotion, University of Jyväskylä, PL 35, FI-40014 Jyväskylä, Finland; 2 UKK Institute of Health Promotion Research, Kaupinpuistonkatu 1, FI-33500 Tampere, Finland; 3 Faculty of Medicine and Health Technology, Tampere University, Tampere, Finland; 4 Paavo Nurmi Centre & Unit for Health and Physical Activity, University of Turku, Kiinamyllykatu 10, FI-20520 Turku, Finland; 5 Medical Research Center (MRC), University of Oulu and University Hospital of Oulu, Oulu, Finland; 6 Department of Sports and Exercise Medicine, Oulu Deaconess Institute Foundation sr, P.O. Box 365, FI-90101 Oulu, Finland; 7 Center for Life Course Health Research, University of Oulu, Oulu, Finland; 8 Department of Mathematics and Statistics, University of Jyväskylä, P.O. Box 35, Jyväskylä, Finland; 9 Tampere Research Center of Sports Medicine, Kaupinpuistonkatu 1, FI-33500 Tampere, Finland; 10 Department of Clinical Physiology and Nuclear Medicine, Kuopio University Hospital, Kuopio, Finland; 11 Kuopio Research Institute of Exercise Medicine, Haapaniementie 16, FI-70100 Kuopio, Finland; 12 Clinic for Sports and Exercise Medicine, Foundation for Sports and Exercise Medicine, Alppikatu 2, FI-00530 Helsinki, Finland; 13 Department of Sports and Exercise Medicine, Clinicum, University of Helsinki, Helsinki, Finland; 14 Research Center for Olympic Sports, Rautpohjankatu 6, FI-40700 Jyväskylä, Finland

**Keywords:** Physical activity, Sports, Adolescence, Young adults, Longitudinal studies, Sedentary behaviour, Accelerometer

## Abstract

**Background:**

Longitudinal studies demonstrate an average decline in physical activity (PA) from adolescence to young adulthood. However, while some subgroups of adolescents decrease activity, others increase or maintain high or low activity. Activity domains may differ between subgroups (exhibiting different PA patterns), and they offer valuable information for targeted health promotion. Hence, the aim of this study was to identify PA patterns from adolescence to young adulthood; also to explore the associations of (i) changes in PA domains and in sedentary time, (ii) sociodemographic factors, and (iii) self-rated health with diverging PA patterns.

**Methods:**

The observational cohort study data encompassed 254 adolescents at age 15 and age 19. K-means cluster analysis for longitudinal data was performed to identify participant clusters (patterns) based on their accelerometry-measured moderate-to-vigorous PA (MVPA). Logistic regressions were applied in further analysis.

**Results:**

Five PA patterns were identified: *inactivity maintainers* (*n* = 71), *activity maintainers* (*n* = 70), *decreasers from moderate (to low) PA* (*n* = 61), *decreasers from high (to moderate) PA* (*n* = 32), and *increasers* (*n* = 20).

At age 15, participation in sports clubs (SC, 41–97%) and active commuting (AC, 47–75%) was common in all the patterns. By age 19, clear dropout from these activities was prevalent (SC participation mean 32%, AC 31–63%). Inactivity maintainers reported the lowest amount of weekly school physical education.

Dropout from SC – in contrast to non-participation in SC – was associated with higher odds of being a decreaser from high PA, and with lower odds of being an inactivity maintainer. Maintained SC participation was associated with higher odds of belonging to the decreasers from high PA, and to the combined group of activity maintainers and increasers; also with lower odds of being an inactivity maintainer. Maintenance/adoption of AC was associated with decreased odds of being an inactivity maintainer. Self-reported health at age 19 was associated with the patterns of maintained activity and inactivity.

**Conclusions:**

PA patterns diverge over the transition to adulthood. Changes in SC participation and AC show different associations with diverging PA patterns. Hence, tailored PA promotion is recommended.

**Supplementary Information:**

The online version contains supplementary material available at 10.1186/s12966-021-01130-x.

## Background

Longitudinal studies have shown that physical activity (PA) declines from adolescence to young adulthood [1]. These results, which are based on *population-level averages*, mask individual variation, with some subgroups of young people increasing or decreasing PA, and others maintaining (in)activity. In recent years, it has thus become more common to identify distinct PA patterns^1^ in efforts to gain more specific information on PA evolution over time [2, 11, 12]. For example, using accelerometry to measure moderate-to-vigorous PA (MVPA) at ages 5–19, Kwon et al. [13] identified four trajectories, namely *consistently inactive* (15%), *consistently active* (18%), *decreasing moderate PA* (53%), and *substantially decreasing high PA* (14%). In some studies, a subgroup of PA *increasers* has been further identified, with a prevalence varying between 7 and 14% [2, 3, 7].

It is important to identify PA development over time and to further characterize PA patterns in order to gain more information for targeted and evidence-informed health promotion [4, 10, 11, 14]. Determinants at many levels – including individual, social, environmental, and policy-related – may predict PA change over time [15] and e.g. declined PA may be explained by different factors from those relevant to sustained inactivity [11]. Information on these differing determinants may be useful in planning PA promotion interventions while taking into account the distinct forms of PA evolution during the transition to young adulthood. So far, studies have been conducted on the associations of various determinants and correlates with PA change patterns during adolescence, focusing on e.g. BMI [3, 7, 12], maturity [12], health behaviours [3, 7], parental PA [3], education [3, 12], socioeconomic status [16], and support for PA [7].

Considered as a PA domain (i.e. the context in which PA occurs) [17], *organized sports participation* has been related to different PA patterns [3, 13]. However, only a few prospective studies have explored the simultaneous evolution of several domains of PA over time [17, 18], and no studies have aimed to assess comprehensively the multiple domains of PA and the changes within them in order to identify differences between PA patterns (e.g. observing whether withdrawal from active commuting (AC) and sports club (SB) participation is associated with a pattern of declining activity). Nor has measured sedentary time been included in the analyses of the determinants and correlates of PA change patterns. It can be claimed that both PA and sedentary behaviour measurements are needed, bearing in mind that young people can engage in considerable sedentary time and PA on the same day [19].

So far, few studies have examined PA change and maintenance during the transition to young adulthood on the basis of measured PA rather than self-reported PA [1, 11]. The aims of this longitudinal study were:

1. to identify *PA change patterns* from adolescence to young adulthood (also characterizing the groups further by distributions of different intensities of PA and sedentary behaviour),
2. to explore how changes in PA domains and in sedentary time are associated with diverging PA patterns. (The domains analysed were *sports club participation, active commuting*, and the *amount of school physical education* (assessed only at age 15)).
3. to examine how sociodemographic factors and self-rated health are associated with diverging PA patterns.

## Methods

### Study sample

This observational cohort study encompassed adolescents who participated in the *Health Promoting Sports Club* (HPSC) study at ages 15 (i.e. in 2013–2014) [20] and 19 (i.e. as young adults, in 2017–2018). At baseline, the participants were recruited from (i) 156 sports clubs representing the ten most popular sports in Finland; (ii) 100 schools (pupils both with and without SC participation) from six large cities and surrounding communities in different parts of Finland [See Additional file 1, described in detail in [20]].

The recruited individuals initially completed two internet surveys on health behaviour and on musculoskeletal health. Pre-participation screening was then conducted on randomly selected respondents based on power calculations (described in [20]). The screening, which was done in one of the six national Sports and Exercise Medicine Centres of Excellence, included screening by a physician, and a fasting blood sample [20]. Instruction was also given on the use of a hip-worn accelerometer, including guidance on wearing the device during waking hours for seven consecutive days, except during shower or water activities. Two-thirds (65%) of those participating in the baseline pre-participation screening at age 15 (*n* = 590) participated also in the follow-up screening at age 19 (*n* = 371). After excluding swimmers (as they were unable to use the accelerometer during their swimming training), 254 adolescents provided valid accelerometer data and written consent for both measurement points.

### Measures

The PA and sedentary time of the study participants were measured via a Hookie accelerometer (AM20 Activity Meter, Hookie Technologies Ltd., Helsinki, Finland), which has been shown to be a valid measurement tool among both young persons [21] and adults [22, 23]. The accelerometer collected and stored tri-axial data as actual g-units with a 100 Hz sampling frequency. The data were analysed in units of 6 s’ duration. The PA analysis was based on mean amplitude deviation analyses (MAD), calculated from a resultant tri-axial raw acceleration signal, and converted to metabolic equivalents (METs) [22, 23]. The accuracy of the energy consumption estimation for the MAD method is about 1.2 MET for bipedal locomotion over a wide range of speed. Light PA was defined as an MET value higher than or equal to 1.5, and less than 3.0 (with an MAD value of 22.5–91.5 mg), moderate PA as an MET value higher than or equal to 3.0 and less than 6.0 (91.5 mg–414 mg), and vigorous PA as an MET value higher than or equal to 3.0 (MAD over 414 mg) [23]. In further analyses, the values for moderate and vigorous activity were combined to form an MVPA group. Further details of the device measurements are presented in Additional file 2.

In line with the definition of sedentary behaviour [24], separate analyses were conducted on activity without movement under 1.5 MET while (i) seated or in a reclining/lying posture, and (ii) while standing. Determination of the body posture was based on two facts, namely that the earth’s gravity vector is constant and that the body posture during walking is upright. The accelerometer orientation (in terms of the gravity vector during walking) was taken as the reference, and the angle for posture estimation (APE) was determined from the incident accelerometer orientation in relation to the reference vector [21]. If the total accelerometer wear-time exceeded 18 h per day, hours in excess of 18 were decreased from the sedentary time, as this indicated that the individual was wearing the device during sleep (1.3% of the measurement days).

The PA and sedentary time variables are reported as proportions of valid accelerometer wear-time, and MVPA as mean daily hours and minutes. In addition, we formulated a variable denoting *change in percentage of sedentary time* between baseline and follow-up measurements (based on the difference between baseline and follow-up in the percentage of device wear-time in sedentary positions, denoting either increasing or decreasing sedentary time).

*Participation in SC* was confirmed by questions in three separate surveys (e.g. ‘Are you participating in SC activities?’ with response options ‘Yes*’* and ‘No*’,* and ‘How many times during a normal week do you participate in coach-led training?’) together with a training diary. Based on the categorization of SC participants and non-participants at baseline and follow-up, the *change in SC participation* was formed via a variable comprising four groups: (i) *never participated in SC*; (ii) *dropped out of SC*; (iii) *adopted SC participation*; and (iv) *maintained SC participation*. Because of the small number of cases in (iii) *adopted SC participation* (*n* = 2), categories (iii) and (iv) were combined into a single category, i.e. *maintained or adopted SC participation.*

*AC* to school (since the subjects were 15-year-olds) was estimated via the question ‘How do you commute on your way to and from school at this time of year (select the single most common option)’. The corresponding question among 19-year-olds was assessment of AC to work or to study facilities. Commuters were categorized as ‘active’ (walking and cycling) or ‘non-active’ (those who selected any other response option, such as ‘by car’). Based on this categorization at baseline and at follow-up, a variable denoting *change in AC* was formulated: (i) *never commuted actively*; (ii)) *stopped AC*; (iii) *adopted AC*; and (iv) *maintained AC*. Due to the small number of cases in (iii) *adopted AC (n* = 27), categories (iii) and (iv) were combined in the analysis within a single category, i.e. *maintained or adopted AC*.

*Amount of school physical education* (at age 15) was estimated via the question ‘How many minutes of school physical education do you have in a week? (calculate class physical education plus any optional physical education you do at school)’.

### Statistical analysis

To form different PA change and stability groups (encompassing PA patterns), an analysis was performed using a k-means algorithm for longitudinal data (KmL) [25]. The method grouped observations of physical activity (MVPA) at the two measurement points (ages 15 and 19) into homogeneous subgroups (i.e. clusters that were as heterogeneous as possible from each other). KmL has been shown to have good clustering performance, especially when the sample size or number of measurement points is small [26].

The KmL assigns each observation to a cluster, and the optimal solution is reached by altering two phases: (i) determining the centre of each cluster, and (ii) assigning each observation to its nearest cluster [25]. To find the optimal number of clusters and the best solution we allowed the KmL to run for two to six clusters, 20 times each. The final solution was decided according to the following criteria: (i) Calinski & Harabatz criterion (the optimal number of clusters is the number that maximizes the between-matrix variance and minimizes the within-matrix variance) [25]. As the solutions for four and five clusters were almost equally good, the decision was also based on (ii) the number of participants in each cluster (≥20 observations), and (iii) the amount of MVPA in the most inactive group (taken to be clearly under one hour per day at baseline). This made it possible to obtain meaningful comparisons between groups (bearing in mind that the recommendation for health-enhancing PA for children and adolescents is an average of (at least) 60 min/day of MVPA [27]). It is further in line with the common practice of validating cluster selection also by expert opinion (i.e. reasoning from the subject details) [25, 28].

The differences in frequencies between PA patterns were based on the Chi-square test and on Fisher’s exact test. The Kruskal-Wallis test was used to analyse differences in mean values cross-sectionally (with post hoc Dunn’s test, adjusted by the Bonferroni correction for multiple tests). The significance of the change over time was calculated by the McNemar test and the Wilcoxon signed rank test.

Multivariable binary logistic regression analysis was used to calculate odds ratios (ORs) and 95% confidence intervals (CIs) for the association between the explanatory variables (i) gender, changes in (ii) SC participation, (iii) active commuting, and (iv) percentage of device wear-time by sedentary time, and by membership of each PA group as compared to all the other patterns taken together (representing the outcome). The categories of *activity maintainers* and *increasers* were combined due to the relatively small number of *increasers* (*n* = 20), and because both of the categories represented a favourable evolution of PA in terms of health. The models were adjusted according to *the change in the device wear-time* to eliminate the effect of differences in usage time on the results.

Additional logistic regression analysis was conducted with self-rated health and sociodemographic factors as explanatory variables. Furthermore, linear regression analysis was carried out to illustrate the correlates and determinants for the PA change in the entire sample. In addition to the change in device wear-time and in the season applied in the first measurement, the linear regression analysis was adjusted for baseline MVPA (because the aim was to examine whether explanatory variables could be detected independently of baseline PA levels).

Data analysis was performed using SPSS version 24, except when identifying PA patterns with R package KmL (R software version 3.6.0). The significance level was *p* < 0.05 in all the statistical tests.

## Results

### Characteristics of the study sample

The study sample was 60% female (Table 1), and at baseline 65% of the participants lived in families with high affluence [see Additional file 3]. At post-measurement (age 19) most of the participants (69%) were still living with their parents. Half of the sample (49%) were studying in upper secondary education, while 15% of the participants were studying in higher education, and 21% had started work at post-measurement.

**Table 1:** MVPA and changes in self-reported domains of PA by PA patterns

		Total	PA patterns					***p***^**a**^
			A	B	C	D	E	
*N* (%)		254 (100)	71 (28)	70 (28)	61 (24)	32 (13)	20 (8)	
**Males**, *n* (%)		101 (40)	19 (27)	25 (36)	22 (36)	26 (81)	9 (45)	**< 0.001**
**MVPA mean per day**: hours and minutes (SD)								
age 15		1:22 (0:33)	0:47 (0:12)	1:19 (0:17)	1:25 (0:12)	2:20 (0:24)	1:50 (0:28)	**A < B-E < 0.001** **B < D < 0.001** **B < E 0.004** **C < D < 0.001**
age 19		1:05^b^ (0:34)	0:39 (0:15)	1:25 (0:15)	0:44 (0:11)	1:07 (0:21)	2:25 (0:31)	**A < B,D,E < 0.001** **B > C < 0.001** **B < D 0.037** **B < E < 0.001** **C < D,E < 0.001** **D < E < 0.001**
**Sports club participation**								
age 15, *n* (%)		176 (69)	29 (41)	51 (73)	47 (77)	31 (97)	18 (90)	**< 0.001**
age 19, *n* (%)		97 (38)	9 (13)	33 (47)	23 (38)	19 (59)	13 (65)	**< 0.001**
*p* for sig. Over time		**< 0.001**	**< 0.001**	**< 0.001**	**< 0.001**	**< 0.001**	0.062	
change^a^ *n* (%)	**never**	76 (30)	41 (58)	18 (26)	14 (23)	1 (3)	2 (10)	**< 0.001**
	**dropout**	81 (32)	21 (30)	19 (27)	24 (39)	12 (38)	5 (25)	0.499
	**maintenance**	95 (37)	8 (11)	32 (46)	23 (38)	19 (59)	13 (65)	**< 0.001**
	**adopt**	2 (1)	1 (1)	1 (1)	0 (0)	0 (0)	0 (0)	–
**Active commuting**								
age 15, *n* (%)		142 (57)	32 (47)	40 (58)	35 (57)	20 (67)	15 (75)	0.161
age 19, *n* (%)		76 (30)	17 (24)	25 (36)	20 (33)	6 (19)	8 (42)	0.228
*p* for sig. Over time		**< 0.001**	**0.009**	**0.009**	**0.012**	**0.008**	**0.039**	
change^a^ *n* (%)	**never**	78 (32)	30 (49)	22 (36)	18 (34)	5 (20)	3 (17)	**0.036**
	**dropout**	94 (38)	21 (31)	22 (32)	23 (38)	19 (63)	9 (47)	**0.024**
	**maintenance**	47 (19)	10 (16)	18 (29)	12 (23)	1 (4)	6 (33)	**0.042**
	**adopt**	27 (11)	6 (9)	7 (10)	8 (13)	5 (17)	1 (5)	0.732
**School physical education** at age 15, mean minutes (*n*)		135 (239)	108 (66)	144 (66)	143 (59)	139 (28)	170 (20)	**A < B 0.022** **A < C 0.017** **A < E 0.001**

A = Inactivity maintainers, B = Activity maintainers, C = Decreasers from moderate PA, D = Decreasers from high PA, E = Increasers, MVPA = moderate-to-vigorous physical activity, PA = physical activity

^a^ *p*-values have been calculated comparing the frequency of the individual response category to each of the corresponding response categories for the other PA patterns (e.g. dropout vs. no dropout (= never+maintenance+adopt))

^b^ Significance over time *p* < 0.001. At age 15: 1 h 22 min = 9.7% of device wear-time; at age 19: 1 h 5 min = 7.9% of device wear-time

Note: *p*-values have been assessed using the Chi-square test or Fisher exact test (in cases of sparse data) for categorical variables. The Kruskal-Wallis test was used in analysing differences in mean values between PA patterns cross-sectionally (post hoc Dunn’s test adjusted by the Bonferroni correction for multiple tests); the McNemar test and the Wilcoxon Signed Rank test were used to analyse differences over time

There were no differences in baseline MVPA, perceived health, family affluence, or self-reported PA between the study participants and those lost to follow-up [see Additional file 4]. Males and those who reported lower school achievement (*p* < .001), were more likely not to participate in the post-measurement.

### Patterns of PA

**Fig. 1:**
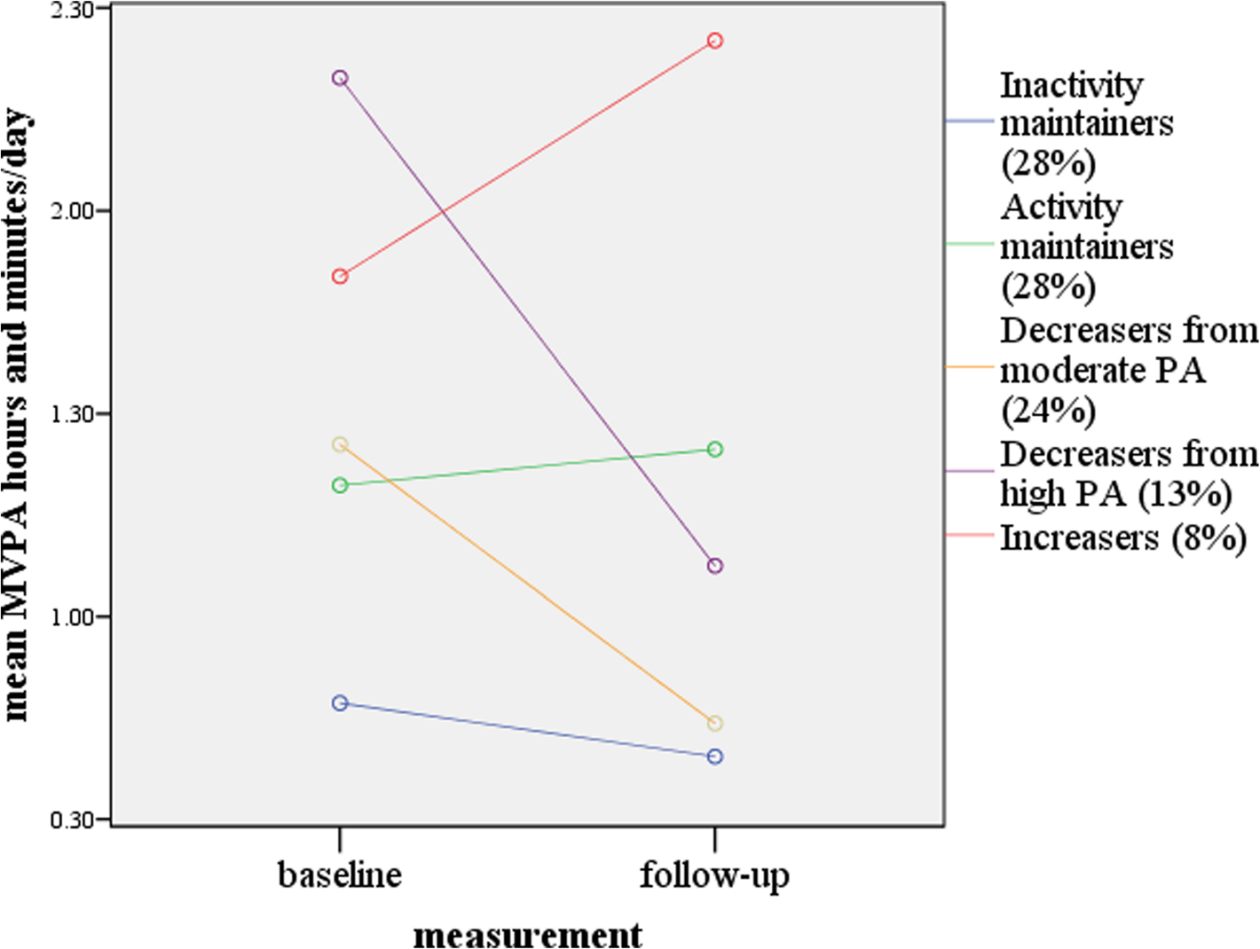
Measured PA patterns (formed by the KmL data-driven clustering method [23]) (*n* = 254)

Five distinct PA change patterns were identified (Fig. 1), concurrently with an overall decline in participants’ (*n* = 254) mean MVPA (Table 1). The most prevalent patterns were *inactivity maintainers* (*n* = 71) and *activity maintainers* (*n* = 70). Two distinct declining PA patterns were found: *decreasers from moderate* (to low) *PA* (*n* = 61), and *decreasers from high* (to moderate) *PA* (*n* = 32). The analysis also revealed a small group of *increasers* (*n* = 20), whose mean daily MVPA was already at a high level at age 15 (1 h 50 min) (Table 1).

### Distribution of different intensities of PA and sedentary behaviour among the PA patterns

**Fig. 2:**
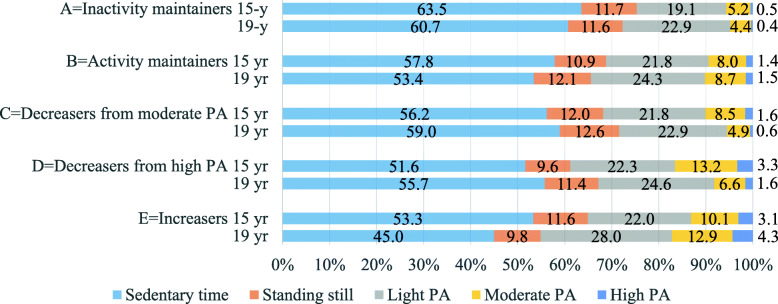
Sedentary behaviour and physical activity (PA) as proportions of device wear-time by PA pattern. Sedentary time 15-y.: A > B**, C-E***, D < B**; 19-y.: E < D*, C, A***, B < C**, A***, change over time: B***, C**, D, E*. Standing still 15-y.: D < C*. Light PA 15-y.: A < B, D*, C**; change over time: A***, B**, E*. Moderate PA 15-y: A < B-E***, D > B, C***; 19-y.: A < B, D, E***, C < D*, C < B, E***, D < B**, E***; change over time: A, E*, C, D***. High PA 15-y: A < B-***, B < E**, D***, C < D**; 19-y.: A < B-E***, C < B, E***, D**; change over time: A*, C, D***. *p*-values determined from the Kruskal-Wallis test for differences in mean values between PA patterns cross-sectionally (post hoc Dunn’s test adjusted by the Bonferroni correction for multiple tests) (only significant differences presented) and the significance of the changes over time in mean values by the Wilcoxon Signed rank test. *p < 0.05, **p < 0.01, ***p < 0.001

Figure 2 illustrates the distribution of different intensities of PA among the PA change patterns. *Inactivity maintainers* spent a higher proportion of their waking hours in sedentary behaviour at age 15 (63.5%) as compared to the other PA patterns (51.6–57.8%), and at age 19 (60.7%), as compared to (at age 19) *activity maintainers* (53.4%) and *increasers* (45.0%) (Fig. 2). Standing still encompassed 10–13% of waking hours among all the patterns, and no changes over time were observed.

The time spent in light PA increased over time among *inactivity and activity maintainers*, and also among *increasers* (Fig. 2). Hence, total PA slightly increased among *inactivity maintainers (p* = .009), even if the corresponding proportion of MVPA decreased at the same time. However, the proportion of total PA among *inactivity maintainers* at age 19 (28%) remained lower as compared to *activity maintainers* (35%), *decreasers from high PA* (33%), and *increasers* (45%).

### Domains of PA by PA patterns

At age 15, SC participation and AC were prevalent in all the patterns (Table 1). Only SC participation differed by pattern (*p* < .001), varying from 41% of *inactivity maintainers* to 97% of *decreasers from high PA* at age 15. On average 57% of participants commuted actively to school.

SC participation and AC decreased over time among all the patterns, with the exception of SC participation among *increasers* (*p* < .062) (Table 1). The prevalence of dropout from SC did not vary with the pattern (*p* = .499, mean 32%). Withdrawal from AC was lowest (at 31%) among *activity maintainers*, and highest (at 63%) among *decreasers from high PA* (*p* = .024).

*Inactivity maintainers* reported less school physical education per week (mean 108 min), as compared to *activity maintainers* (144 min), *decreasers from moderate PA* (143 min), and *increasers* (170 min) (Table 1).

### Simultaneous associations of changes in SC participation, AC, and sedentary time with PA patterns

Dropping out of SC (OR = 0.3, CI = 0.2–0.6) and maintained/adopted SC participation (OR = 0.1, CI = 0.02–0.2) – as opposed to ‘never’ participation in SC – were associated with lower odds of belonging to *inactivity maintainers* as compared to the other PA patterns (Table 2). Correspondingly, *maintained/adopted SC participation* – as opposed to ‘never’ participation in SC (OR = 3.6, CI = 1.8–7.4) – was associated with higher odds of belonging to the combined group of *activity maintainers and increasers*, as compared to the other PA patterns. Moreover, dropping out of SC (OR = 10.9, CI = 1.3–90.7) and maintenance/adoption of SC participation (OR = 11.2, CI = 1.4–90.0) – as opposed to ‘never’ participation in SC – were significant predictors for belonging to *decreasers from high PA*.

**Table 2:** Logistic regression models for physical activity patterns

	Inactivity maintainers		Activity maintainers + increasers		Decreasers from moderate PA		Decreasers from high PA	
	OR (95% CI)	***p***	OR (95% CI)	***p***	OR (95% CI)	***p***	OR (95% CI)	***p***
Gender								
Female	1.0		1.0		1.0		1.0	
Male	0.5 (0.2–1.01)	0.055	0.8 (0.4–1.4)	0.376	0.8 (0.4–1.5)	0.400	**7.4 (2.6–21.3)**	**< 0.001**
Sports club participation								
Maintenance or adopt	**0.1 (0.02–0.2)**	**< 0.001**	**3.6 (1.8–7.4)**	**< 0.001**	1.5 (0.7–3.3)	0.339	**11.2 (1.4–90.0)**	**0.023**
Withdrawal	**0.3 (0.2–0.6)**	**0.001**	1.2 (0.6–2.5)	0.625	1.9 (0.9–4.3)	0.097	**10.9 (1.3–90.7)**	**0.027**
Never	1.0		1.0		1.0		1.0	
Active commuting								
Maintenance or adopt	**0.3 (0.1–0.7)**	**0.004**	1.7 (0.8–3.4)	0.165	1.5 (0.7–3.2)	0.343	1.3 (0.3–4.7)	0.744
Withdrawal	0.5 (0.2–1.03)	0.061	1.0 (0.5–2.0)	0.989	1.2 (0.5–2.5)	0.708	2.3 (0.8–7.3)	0.144
Never	1.0		1.0		1.0		1.0	
Change in % of device wear-time by sedentary time	0.99 (0.96–1.02)	0.359	**0.96 (.93–.98)**	**0.001**	**1.05 (1.01–1.07)**	**0.004**	**1.04 (1.001–1.083)**	**0.046**
Model statistics:								
R^2^ Nagelkerke	0.324		0.179		0.113		0.347	
R^2^ Cox&Snell	0.223		0.131		0.076		0.182	
Hosmer&Lemeshow	0.148		0.631		0.241		0.998	

Note: Adjusted for change in the device wear-time. Statistically significant odds ratios are in bold. Binary analysis: separately for each pattern vs. all the others together. PA = physical activity

Decreased sedentary time (OR = 0.96, CI = 0.93–0.98) was associated with higher odds of being in the combined group of *activity maintainers and increasers* as compared to other PA patterns (Table 2). Correspondingly, an increased sedentary time was related to higher odds of being a *decreaser from moderate PA* (OR = 1.05, CI = 1.01–1.07) and *decreaser from high PA* (OR = 1.04, CI = 1.0–1.1).

Male gender was associated with increased odds of being a *decreaser from high PA* (OR = 7.4, CI = 2.6–21.3) (Table 2). Maintained/adopted AC (OR = 0.3, CI = 0.1–0.7) – as opposed to ‘never’ (at either at age 15 or age 19) participation in active commuting – was associated with lower odds of being an *inactivity maintainer.*

### Sociodemographic characteristics and self-rated health among PA patterns

The analysis in Additional file 3 shows that no differences were observed between PA change patterns in any of the studied sociodemographic variables. However, good self-rated health related to higher odds of belonging to the combined group of *activity maintainers and increasers*, (OR = 2.7, CI = 1.1–6.8) and lower odds of being an *inactivity maintainer* (OR = 0.4, CI = 0.2–0.9) [see Table 3 and 1 in Additional file 5].

## Discussion

Despite a general declining tendency in mean MVPA (see also [1]), the evolution of PA from adolescence to young adulthood varied greatly. The PA analysis identified five distinct PA patterns. Half of the study population maintained their activity or inactivity, while two groups with declining PA were observed, starting from a different baseline MVPA level (high or moderate). There was also a small group of increasers, who were already highly active at baseline (age 15). Furthermore, the changes in SC participation, AC, and sedentary time showed different relationships with the PA patterns. The results support the use of tailored PA promotion for the different subgroups.

Previous studies identifying longitudinal PA patterns during adolescence [2–4, 7, 11] have varied in the amount and prevalence of patterns, the characteristics of the study sample (e.g. size and number of measurement points), methods for PA measurement, and cluster identification. Any subgroup of increasers detected has been small, with a prevalence varying between 7 and 12% [2, 3, 7]. This is consistent with the findings of the present study.

*Inactivity maintainers* turned out to be a group in which *all* the examined PA domains were less likely to occur. In this group, inactivity appeared in many forms: in a lower level of school physical education – indicating a lower selection of optional physical education classes – and a higher risk of non-participation in SC and AC as compared to the other PA groups. Promotion of any of the studied PA domains might have potential for increasing total PA. However, PA is a complex behaviour, and an increase in one PA domain might result in a decrease in another domain.

An important finding was that AC prevalence declined in all the groups (with dropout prevalence varying from 31 to 63%), even if the AC change significantly differentiated only the *inactivity maintainers* from the rest of the study population. Previous studies have found either a declining or a stable AC trend among young people on average [17]. Interestingly, AC maintenance during adolescence has been found to predict later midlife PA [29]. There seems good reason to encourage young people to maintain or adopt active forms of transport – including in post-school years – at least when the distances involved make AC feasible.

It is notable that as many as 41% of the *inactivity maintainers* were participating in SC at age 15. The result implies that SC participation does not guarantee sufficient PA for all adolescents, in terms of the recommendations for health-enhancing PA (i.e. at least one hour per day on average) [27]. Corresponding results have previously been found in a sample of SC participants [30]. However, one should take into account that the population in the present study represents young people who are more active than the average in Finland; in fact the mean daily MVPA was eight minutes more than that found in a population-based study [31]. Moreover, the *inactivity maintainers* in the present study were relatively active (mean daily MVPA = 47 min at age 15). In some previous studies, participants in the ‘consistently inactive’ trajectory have been clearly less active, with a mean daily MVPA of less than 30 min at age 15 [2, 10, 12, 13, 32]. One of those studies also explored participation in sports, and *all* of the subjects in the most inactive pattern also followed a trajectory of no participation in sports [13].

Despite the above, the message of this study is clear. SC participation was associated with a sustained or increased PA pattern, and correspondingly, SC withdrawal with a PA decrease from a high level. The relationship between sports participation at adolescence and PA in later life has been noted in previous studies [3, 33]. Although SC participation showed a range of associations with PA patterns, dropout from SC was common in all the patterns (mean 32%). In terms of PA promotion, the challenges are two-fold: how to prevent dropout from SC, and how to support compensatory PA as a substitute for SC activities, in cases where SC participation has come to an end during young adulthood.

Male participants were more likely to be present in the group exhibiting declining PA from a high baseline level. A review of previous studies found a slightly larger decrease in males than in females from adolescence to adulthood, possibly due to higher activity levels in youth [1]. The present study also indicated that even if PA declined from high PA levels, most of the young people in this group were still physically active, since they actually fulfilled the PA guidelines for children and adolescents at age 19 [27].

*Decreasers from moderate PA* formed the only group in which, at age 19, the activity fell to approximately the same level as that of the *inactivity maintainers*. Furthermore, as compared to the other patterns, the pattern of PA decrease from a moderate to a low level was not predicted by gender, or by sociodemographic factors; nor was it related to certain types of changes in SC participation or AC. Withdrawal from AC and SC between ages 15 and 19 was common overall. It amounted to over one third among *decreasers from moderate PA*, but as the development was *on average* the same in the rest of the study population, no difference was found relative to the other patterns. Future research will be needed to examine other possible determinants and correlates that could characterize this pattern of declining activity. Moreover, more research in general is called for regarding longitudinal PA patterns and their determinants [10, 12, 14]; these might include social and environmental factors, health behaviour, and life changes [11]. If the aim in future is to achieve a deeper understanding of the changes in PA domains over time, various up-to-date methods will be applicable, including GPS [34].

This study indicated that changes in MVPA reflected changes in sedentary behaviour, with increased sedentary time being more likely to occur within the declining PA patterns. Although the result seems somewhat self-evident, it emphasizes the importance of seeking possibilities to discourage sedentary behaviour, as a means to prevent PA decrease. However, among the *inactivity maintainers*, light PA increased at the same time as MVPA slightly decreased – though without any changes in sedentary time. On this basis, one cannot actually claim that sedentary time and MVPA directly displace one another (see also [19]).

Interestingly none of the studied sociodemographic variables related to PA change patterns. The patterns did not even differ in terms of family affluence, despite findings in some previous studies indicating that family socioeconomic factors could explain differences between PA change patterns [3, 16]. Many typical life changes had not occurred at age 19, given that most of the participants were still living with their parents. Only a minority had started studying in higher education or embarked on working life. Hence, in future studies, possible differences in life events and choices between distinct PA change patterns should be examined. Furthermore, differences in the health and health behaviour of young adults in terms of PA patterns will be an important research topic in future, bearing in mind that the results of self-reported health differentiated between PA patterns.

The following limitations should be noted: the study sample was relatively small and does not represent the entire Finnish population of the age cohort in question, even though it was collected from different parts of Finland. Participation in terms of gender and school achievement was unequal. Rather more winter measurements were conducted during the first data collection period as compared to the second data collection; however, difference was taken into account in the analysis. Moreover, partly because of the sampling method (involving both sports clubs and schools), the young people represented by the data are more active than on average; however, this could also be regarded as a strength, insofar as the data enable thorough analysis of high PA maintenance and SC participation. One should also note that additional PA measurement periods would have strengthened the validity of the results, with possibilities for different PA change patterns to be identified between the baseline and post-measurement.

The main strength of the present study is the provision of longitudinal, objectively measured PA data from the (less often studied) period from adolescence to young adulthood (see also [1, 11, 35]), over a diverse sample of Finnish young people. The data-driven extraction of longitudinal PA patterns (KmL) [25] represents a modern way to research change and stability in PA behaviour over time. It should be noted that a linear regression analysis for the entire sample is unable to illustrate the differences in determinants and correlates between distinct PA change patterns [see Additional file 6]. This highlights the importance of pattern-based analysis. Another novel aspect is the analysis of multiple PA domains in relation to PA patterns, taking into account the change in both outcome (PA patterns) and explanatory variables. Former prospective studies on changes in PA domains have rarely included several domains in the same sample [17].

## Conclusions

Five distinct PA change and stability patterns could be identified. Dropout from SC and from AC was prevalent in all the PA patterns. The study highlights the importance of SC participation in supporting maintenance of PA, and preventing a decrease in PA. An absence of AC and of SC participation was associated with sustained inactivity from adolescence to young adulthood. The group of inactivity maintainers also reported a lower level of (optional) school physical education as compared to the other PA patterns. In tackling sustained inactivity in the youth population, actions that increase any of the studied PA domains may be appropriate. Preventing dropout from organized sports, and supporting substitutes for SC participation, may be appropriate for preventing a decrease in PA. Similarly, to sustain sufficient activity, young people who up to now have followed favourable PA patterns may benefit from further supportive actions. However, these assumptions need to be tested in intervention studies tailored towards particular PA change and stability patterns. The influence of psychosocial and environmental factors in explaining differences between PA change patterns remains to be investigated in future research.

## Supplementary Information

**Additional file 1.** Flow of participants in this study: persons who participated in pre-participation screening for the Health Promoting Sports Club (HPSC) study and who had valid accelerometer data from both measurement points.

**Additional file 2.** Device-measurement periods by PA change patterns.

**Additional file 3.** Sociodemographic characteristics and self-rated health of the participants, by PA change patterns [36–38].

**Additional file 4.** The loss to the follow-up analysis [36, 37].

**Additional file 5. **Associations of sociodemographic factors and measurement season with physical activity change patterns via logistic regression analysis.

**Additional file 6.** Associations between background variables and change in moderate-to-vigorous physical activity from age 15 to 19.

## Data Availability

The datasets generated and/or analysed during the current study are not publicly available, since they contain confidential personal details and health information but are available from the corresponding author on reasonable request.

## Abbreviations

AC: active commuting
CI: confidence interval
MVPA: moderate-to-vigorous physical activity
OR: odds ratio
PA: physical activity
SC: sports club

## Footnotes

1 The terminology in the field is inconsistent. PA change/maintenance has been presented via *patterns* [2, 3], *trajectories* (especially in studies using trajectory modelling [4–6], but also in studies using other methods [7, 8]), *profiles* [6], *groups* [2, 9], *subgroups* [10], and *clusters* [2], all referring to the same population distributions on the basis of their PA change or maintenance over time.
