# Supplementary figures and images for: Physical activity from adolescence to young adulthood: patterns of change, and their associations with activity domains and sedentary time
Source: Int J Behav Nutr Phys Act. 2021 Jun 30;18:85. doi: 10.1186/s12966-021-01130-x (PMC8246658; doi:10.1186/s12966-021-01130-x)

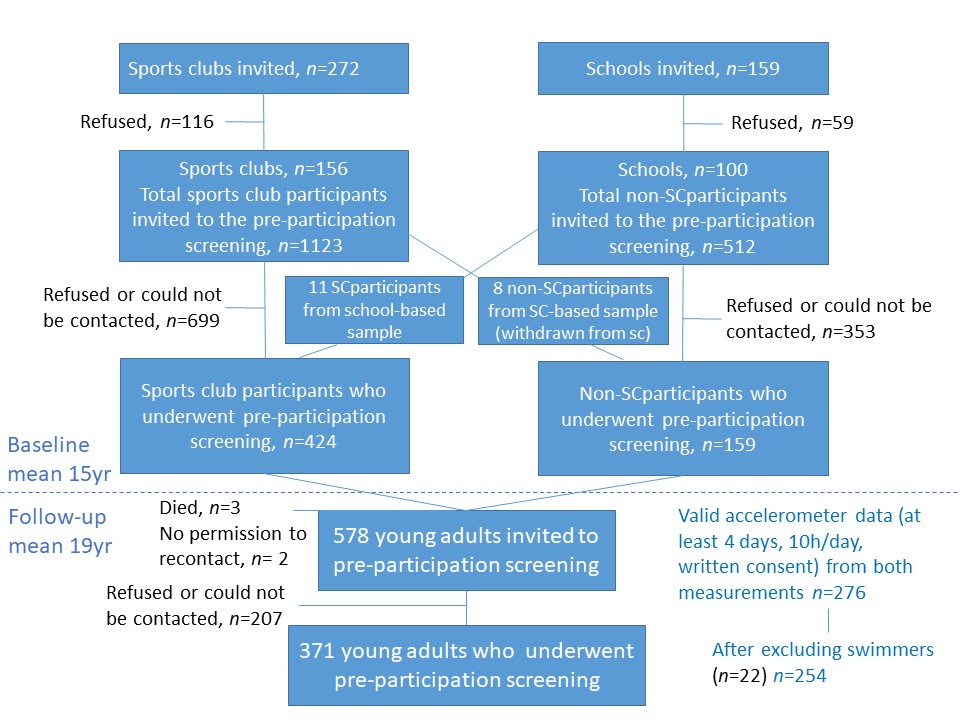

Supplement: Supplementary file 1 — Additional file 1. Flow of participants in this study: persons who participated in pre-participation screening for the Health Promoting Sports Club (HPSC) study and who had valid accelerometer data from both measurement points. [file 12966_2021_1130_MOESM1_ESM.jpg]
