# Supplementary material for: Physical activity from adolescence to young adulthood: patterns of change, and their associations with activity domains and sedentary time
Source: Int J Behav Nutr Phys Act. 2021 Jun 30;18:85. doi: 10.1186/s12966-021-01130-x (PMC8246658; doi:10.1186/s12966-021-01130-x)
Supplement: Supplementary file 2 — Additional file 2. Device-measurement periods by PA change patterns. [file 12966_2021_1130_MOESM2_ESM.docx]

|  | All | Inactivity maintainers | Activity maintainers | Decreasers from  moderate PA | Decreasers from high PA | Increasers | *p* |
| --- | --- | --- | --- | --- | --- | --- | --- |
| Measurement periodᵃ in winter  (Nov-Apr), *n* (%) |  |  |  |  |  |  |  |
| 1^st^ measurementᵃ | **180 (71)** | **59 (83)** | **51 (73)** | **41 (67)** | **17 (53)** | **12 (60)** | **0.020** |
| 2^nd^ measurementᵇ | 130 (51) | 38 (54) | 32 (46) | 36 (59) | 16 (50) | 8 (40) | 0.476 |
| Measurement days, mean (SD) |  |  |  |  |  |  |  |
| 1^st^ measurement | 6.3 (0.86) | 6.4 (0.77) | 6.3 (0.88) | 6.3 (0.95) | 6.3 (0.83) | 6.7 (0.80) | 0.252 |
| 2^nd^ measurement | 6.3 (0.91) | 6.4 (0.83) | 6.2 (0.97) | 6.3 (0.90) | 6.2 (0.998) | 6.6 (0.94) | 0.342 |
| Device wear-time per day,  mean (hours & minutes) |  |  |  |  |  |  |  |
| 1^st^ measurement | 14:04 | 13:58 | 14:05 | 14:10 | 14:04 | 14:01 | 0.763 |
| 2^nd^ measurement | 13:45 | 13:38 | 13:56 | 13:30 | 13:43 | 14:12 | 0.084 |
| *p* for sig. over time | **<0.001** | **0.030** | 0.205 | **<0.001** | 0.112 | 0.411 |  |

Note: Statistically significant *p*-values are in bold; *p*-values have been assessed using Chi-square test or Fisher exact test (in cases of sparse data) for categorical variables. The Kruskal-Wallis test was used in analysing differences in mean values between PA patterns cross-sectionally (post hoc Dunn’s test adjusted by the Bonferroni correction for multiple tests); the Wilcoxon Signed Rank test was used to analyse differences over time.

ᵃ The data for the 1^st^ measurement period were collected from February to May 2013, and from September 2013 to May 2014.

ᵇ The data for the 2^nd^ measurement period were collected from January to June 2017, and from August 2017 to May 2018.
