# Supplementary material for: Physical activity from adolescence to young adulthood: patterns of change, and their associations with activity domains and sedentary time
Source: Int J Behav Nutr Phys Act. 2021 Jun 30;18:85. doi: 10.1186/s12966-021-01130-x (PMC8246658; doi:10.1186/s12966-021-01130-x)
Supplement: Supplementary file 3 — Additional file 3. Sociodemographic characteristics and self-rated health of the participants, by PA change patterns [36–38]. [file 12966_2021_1130_MOESM3_ESM.docx]

**Additional file 3.**

|  | All | Inactivity maintainers | Activity maintainers | Decreasers from  moderate PA | Decreasers from high PA | Increasers | *p* |
| --- | --- | --- | --- | --- | --- | --- | --- |
| Age, mean years (SD) |  |  |  |  |  |  |  |
| 1^st^ measurement | 15.5 (0.6) | 15.6 (0.5) | 15.5 (0.6) | 15.3 (0.5) | 15.5 (0.5) | 15.4 (0.5) | 0.138 |
| 2^nd^ measurement | 19.4 (0.6) | 19.4 (0.7) | 19.4 (0.7) | 19.2 (0.7) | 19.5 (0.6) | 19.5 (0.5) | 0.130 |
| Education and employment status (age 19), *n* (%) |  |  |  |  |  |  |  |
| Studying | 159 (63) | 40 (56) | 47 (67) | 43 (71) | 19 (59) | 10 (53) | 0.743ᵃ |
| in general upper secondary school | 100 (40) | 23 (33) | 29 (41) | 28 (46) | 13 (41) | 7 (37) |  |
| in vocational school | 23 (9) | 9 (13) | 5 (7) | 6 (10) | 2 (6) | 1 (5) |  |
| in higher education institutionᵇ | 39 (16) | 9 (13) | 13 (19) | 11 (18) | 3 (9) | 3 (16) |  |
| Working | 53 (21) | 18 (25) | 13 (19) | 9 (15) | 8 (25) | 5 (26) |  |
| Other | 41 (16) | 13 (18) | 10 (14) | 9 (15) | 5 (16) | 4 (21) |  |
| unemployed/temporarily laid off | 17 (6) | 8 (11) | 3 (4) | 3 (5) | 2 (6) | 1 (5) |  |
| doing military service | 10 (4) | 1 (1) | 5 (7) | 0 | 1 (3) | 3 (16) |  |
| Living with parents (age 19), *n* (%) | 174 (69) | 50 (70) | 48 (69) | 39 (64) | 23 (72) | 14 (74) | 0.892 |
| Living in urban area, *n* (%) |  |  |  |  |  |  |  |
| age 15 | 161 (65) | 35 (52) | 49 (71) | 42 (69) | 21 (70) | 14 (70) | 0.111 |
| age 19 | 198 (78) | 51 (72) | 58 (83) | 49 (80) | 27 (84) | 13 (68) | 0.339 |
| Self-reported school grade average: good to excellent (age 15) (grades 8–10 in grading 4–10), *n* (%) | 205 (83) | 52 (77) | 57 (83) | 52 (85) | 27 (90) | 17 (85) | 0.556 |
| High family affluence^c^ (age15), *n* (%) | 154 (62) | 38 (56) | 44 (64) | 38 (62) | 22 (73) | 12 (60) | 0.589 |
| Self-rated health: good to excellent, *n* (%) |  |  |  |  |  |  |  |
| age 15 | 230 (93) | 61 (90) | 63 (91) | 58 (95) | 28 (93) | 20 (100) | 0.608 |
| age 19 | **214 (85)** | **51 (72)** | **64 (92)** | **51 (84)** | **30 (94)** | **18 (95)** | **0.007** |
| Reached menarche or Tanner stage P4 [31] (age 15), *n* (%) | 242 (95) | 69 (97) | 67 (96) | 57 (93) | 29 (91) | 20 (100) | 0.505 |

Note: Statistically significant *p*-values are in bold; *p*-values have been assessed using Chi-square test or Fisher exact test (in cases of sparse data) for categorical variables. The Kruskal-Wallis test was used in analysing differences in mean values between PA patterns cross-sectionally (post hoc Dunn’s test adjusted by the Bonferroni correction for multiple tests).

ᵃ The *p-*value represents the difference between the groups in bold font (studying, working, other) and PA patterns.

ᵇ University or university of applied sciences

^c^ The assessment of family affluence is based on adolescents’ answers to questions on four common consumption indicators of material deprivation (cars, bedrooms, computers, vacations) [36]. A composite Family Affluence Scale score was calculated for each youth based on his or her responses to these four items. The scale has previously been validated in the Health Behaviour in School-aged Children study [37].
