# Supplementary material for: Physical activity from adolescence to young adulthood: patterns of change, and their associations with activity domains and sedentary time
Source: Int J Behav Nutr Phys Act. 2021 Jun 30;18:85. doi: 10.1186/s12966-021-01130-x (PMC8246658; doi:10.1186/s12966-021-01130-x)
Supplement: Supplementary file 4 — Additional file 4. The loss to the follow-up analysis [36, 37]. [file 12966_2021_1130_MOESM4_ESM.docx]

|  | Study population in this article (*n*=254) | Those who participated in only pre-participation screening at age 15, or who participated also at age 19 but did not have valid accelerometer data (*n*=307) | *p*-valueᵃ |
| --- | --- | --- | --- |
| Females, *n* (%) | 153 (60.2%) | 151 (49.2%) | <0.001 |
| Self-reported school grade average: good to excellent (grades 8–10 in grading 4–10), *n* (%) | 205 (82.7%) | 192 (64.6%) | <0.001 |
| Self-rated health: good to excellent, *n* (%) | 230 (92.7%) | 276 (92.9%) | 0.933 |
| High family affluenceᵇ,  *n* (%) | 186 (62.6) | 214 (62.1) | 0.654 |
| MVPA baseline, mean per day | 1 hour 22 minutes | 1 hour 21 minutesᶜ | 0.940 |

ᵃ *p*-values assessed using Chi Square test and Mann Whitney U test.
ᵇ The assessment of family affluence is based on adolescents’ answers to questions on four common consumption indicators of material deprivation (cars, bedrooms, computers, vacations) [36]. A composite Family Affluence Scale score was calculated for each youth based on his or her responses to these four items. The scale has previously been validated in the Health Behaviour in School-aged Children study [38].
**ᶜ** Information was available from 246 adolescents with valid accelerometer data.
