# Supplementary material for: Physical activity from adolescence to young adulthood: patterns of change, and their associations with activity domains and sedentary time
Source: Int J Behav Nutr Phys Act. 2021 Jun 30;18:85. doi: 10.1186/s12966-021-01130-x (PMC8246658; doi:10.1186/s12966-021-01130-x)
Supplement: Supplementary file 5 — Additional file 5. Associations of sociodemographic factors and measurement season with physical activity change patterns via logistic regression analysis. [file 12966_2021_1130_MOESM5_ESM.docx]

**Table 1.** Associations of gender, living area, and self-rated health with PA change patterns.

|  | Inactivity maintainers | | Activity maintainers + increasers | | Decreasers from moderate PA | | Decreasers from high PA | |
| --- | --- | --- | --- | --- | --- | --- | --- | --- |
|  | **OR (95% CI)** | ***p*** | **OR (95% CI)** | ***p*** | **OR (95% CI)** | ***p*** | **OR (95% CI)** | ***p*** |
| Gender | | | | | | | | |
| Female | 1.0 | | 1.0 | | 1.0 | | 1.0 | |
| Male | **0.5 (0.2–0.9)** | **0.025** | 0.7 (0.4–1.2) | 0.168 | 0.9 (0.5–1.7) | 0.800 | **11.9 (4.2–33.9)** | **<0.001** |
| Living area (age 15) | | | | | | | | |
| Urban | **1.0** |  | 1.0 |  | 1.0 |  | 1.0 |  |
| Rural | **2.1 (1.2–3.9)** | **0.015** | 0.7 (0.4–1.2) | 0.214 | 0.8 (0.4–1.4) | 0.383 | 0.76 (0.3–1.9) | 0.548 |
| Self-rated health (age 19) |  |  |  |  |  |  |  |  |
| Fair or poor | **1.0** |  | 1.0 |  | 1.0 |  | 1.0 |  |
| Good or excellent | **0.3 (0.2–0.7)** | **0.005** | **3.1 (1.3–7.4)** | **0.014** | 1.001 (0.4–2.3) | 0.997 | 1.5 (0.3–7.7) | 0.608 |
| Model statistics: | | | | | | | | |
| R² Nagelkerke | 0.164 | | 0.080 | | 0.048 | | 0.265 | |
| R² Cox&Snell | 0.113 | | 0.058 | | 0.032 | | 0.139 | |
| Hosmer&Lemeshow | 0.550 | | 0.800 | | 0.346 | | 0.845 | |

Note: Adjusted for the measurement season (age 15) and change in the device wear-time. Statistically significant odds ratios are in bold. Binary analysis: separately for each pattern (vs. all the others together). PA= physical activity.

**Table 2.** Associations of living area, changes in PA domain, and sedentary time with PA change patterns.

|  | Inactivity maintainers | | Activity maintainers + increasers | | Decreasers from moderate PA | | Decreasers from high PA | |
| --- | --- | --- | --- | --- | --- | --- | --- | --- |
|  | **OR (95% CI)** | ***p*** | **OR (95% CI)** | ***p*** | **OR (95% CI)** | ***p*** | **OR (95% CI)** | ***p*** |
| Gender |  | |  | |  | |  | |
| Female | 1.0 | | 1.0 | | 1.0 | | 1.0 | |
| Male | 0.5 (0.2–1.01) | 0.056 | 0.8 (0.4–1.4) | 0.401 | 0.8 (0.4–1.5) | 0.336 | **7.4 (2.6–21.2)** | **<0.001** |
| Sports club participation |  |  |  |  |  |  |  |  |
| Maintenance or adopt | **0.1 (0.02–0.2)** | **<0.001** | **3.4 (1.6–7.0)** | **0.001** | 1.4 (0.6–3.2) | 0.413 | **11.5 (1.4–93.6)** | **0.023** |
| Withdrawal | **0.3 (0.2–0.7)** | **0.004** | 1.1 (0.5–2.4) | 0.811 | 1.8 (0.8–4.2) | 0.141 | **11. (1.3–94)** | **0.026** |
| Never | 1.0 | | 1.0 | | 1.0 | | 1.0 | |
| Active commuting |  |  |  |  |  |  |  |  |
| Maintenance or adopt | **0.3 (0.1–0.7)** | **0.004** | 1.7 (0.8–3.4) | 0.166 | 1.4 (0.7–3.1) | 0.365 | 1.3 (0.3–4.7) | 0.741 |
| Withdrawal | 0.5 (0.2–1.1) | 0.085 | 0.96 (0.5–2.0) | 0.913 | 1.1 (0.5–2.4) | 0.766 | 2.3 (0.8–7.4) | 0.141 |
| Never | 1.0 | | 1.0 | | 1.0 | | 1.0 | |
| Change in % of device wear-time by sedentary time | 0.99 (0.96–1.01) | 0.326 | **0.96 (0.93–0.98)** | **0.001** | **1.05 (1.01–1.08)** | **0.004** | **1.04 (1.001–1.08)** | **0.045** |
| Living area |  |  |  |  |  |  |  |  |
| Urban | 1.0 |  | 1.0 |  | 1.0 |  | 1.0 |  |
| Rural | 1.5 (0.8–3.1) | 0.217 | 0.8 (0.4–1.4) | 0.398 | 0.9 (0.4–1.7) | 0.627 | 1.1 (0.4–3.0) | 0.805 |
| Model statistics: |  | |  | |  | |  | |
| R² Nagelkerke | 0.331 | | 0.183 | | 0.114 | | 0.348 | |
| R² Cox&Snell | 0.228 | | 0.122 | | 0.077 | | 0.182 | |
| Hosmer&Lemeshow | 0.437 | | 0.624 | | 0.589 | | 0.987 | |

Note: Adjusted for change in the device wear-time. Statistically significant odds ratios are in bold. Binary analysis: separately for each pattern (vs. all the others together). PA= physical activity.

**Table 3.** Associations of self-rated health, changes in PA domain, and sedentary time with PA change patterns.

|  | Inactivity maintainers | | Activity maintainers + increasers | | Decreasers from moderate PA | | Decreasers from high PA | |
| --- | --- | --- | --- | --- | --- | --- | --- | --- |
|  | **OR (95% CI)** | ***p*** | **OR (95% CI)** | ***p*** | **OR (95% CI)** | ***p*** | **OR (95% CI)** | ***p*** |
| Gender |  | |  | |  | |  | |
| Female | 1.0 | | 1.0 | | 1.0 | | 1.0 | |
| Male | 0.6 (0.3–1.2) | 0.147 | 0.7 (0.4–1.3) | 0.219 | 0.8 (0.4–1.5) | 0.405 | **7.1 (2.4–20.1)** | **<0.001** |
| Sports club participation |  |  |  |  |  |  |  |  |
| Maintenance or adopt | **0.1 (0.02–0.2)** | **<0.001** | **3.4 (1.6–7.0)** | **0.001** | 1.5 (0.7–3.3) | 0.347 | **11.1 (1.4-88.8)** | **0.024** |
| Withdrawal | **0.3 (0.2–0.6)** | **0.002** | 1.2 (0.5–2.5) | 0.709 | 1.9 (0.9–4.3) | 0.100 | **10.8 (1.3–89.6)** | **0.028** |
| Never | 1.0 | 1.0 | 1.0 | 1.0 |  |  |  |  |
| Active commuting |  |  |  |  |  |  |  |  |
| Maintenance or adopt | **0.3 (0.1–0.7)** | **0.007** | 1.6 (0.8–3.3) | 0.201 | 1.5 (0.7–3.2) | 0.347 | 1.2 (0.3–4.7) | 0.756 |
| Withdrawal | 0.5 (0.2–1.03) | 0.059 | 1.004 (0.5–2.1) | 0.992 | 1.2 (0.5–2.5) | 0.708 | 2.4 (0.8–7.5) | 0.137 |
| Never | 1.0 | 1.0 | 1.0 |  |  |  |  |  |
| Change in % of device wear- time by sedentary time | 0.99 (0.96–1.02) | 0.331 | **0.96 (0.93–0.98)** | **0.001** | **1.1 (1.01–1.08)** | **0.004** | **1.04 (1.001–1.08)** | **0.047** |
| Self-rated health (age 19) |  |  |  |  |  |  |  |  |
| fair or poor | 1.0 |  | 1.0 |  | 1.0 |  | 1.0 |  |
| good or excellent | **0.4 (0.2–0.9)** | **0.030** | **2.7 (1.1–6.8)** | **0.035** | 1.02 (0.4–2.4) | 0.961 | 1.4 (0.3–7.8) | 0.695 |
|  |  |  |  |  |  |  |  |  |
| Model statistics: |  | |  | |  | |  | |
| R² Nagelkerke | 0.345 | | 0.203 | | 0.113 | | 0.348 | |
| R² Cox&Snell | 0.238 | | 0.148 | | 0.076 | | 0.182 | |
| Hosmer&Lemeshow | 0.876 | | 0.442 | | 0.242 | | 0.898 | |

Note: Adjusted for change in the device wear-time. Statistically significant odds ratios are in bold. Binary analysis: separately for each pattern (vs. all the others together). PA= physical activity.

**Table 4.** Associations of changes in PA domain, and sedentary time with PA change patterns with adjustment for measurement season.

|  | Inactivity maintainers | | Activity maintainers + increasers | | Decreasers from moderate PA | | Decreasers from high PA | |
| --- | --- | --- | --- | --- | --- | --- | --- | --- |
|  | **OR (95% CI)** | ***p*** | **OR (95% CI)** | ***p*** | **OR (95% CI)** | ***p*** | **OR (95% CI)** | ***p*** |
| Gender |  | |  | |  | |  | |
| Female | 1.0 | | 1.0 | | 1.0 | | 1.0 | |
| Male | 0.5 (0.2–0.97) | 0.461 | 0.8 (0.4–0.8) | 0.410 | 0.8 (0.4–1.5) | 0.402 | **9.1 (3.0–27.3)** | **<0.001** |
| Sports club participation |  |  |  |  |  |  |  |  |
| Maintenance or adopt | **0.1 (0.02–0.2)** | **<0.001** | **3.6 (1.7–7.3)** | **0.001** | 1.5 (0.7–3.3) | 0.342 | **11.6 (1.4–95.5)** | **0.023** |
| Withdrawal | **0.4 (0.2–0.8)** | **0.006** | 1.2 (0.5–2.5) | 0.706 | 1.9 (0.9–4.3) | 0.102 | **10.2 (1.2–87.1)** | **0.034** |
| Never | 1.0 | | 1.0 | | 1.0 | | 1.0 | |
| Active commuting |  |  |  |  |  |  |  |  |
| Maintenance or adopt | **0.3 (0.1–0.7)** | **0.003** | 1.7 (0.8–3.4) | 0.161 | 1.5 (0.7–3.2) | 0.345 | 1.6 (0.4–6.3) | 0.482 |
| Withdrawal | **0.4 (0.2–0.97)** | **0.042** | 1.0 (0.5–2.0) | 0.993 | 1.2 (0.5–2.5) | 0.708 | 2.5 (0.8–8.1) | 0.125 |
| Never | 1.0 | | 1.0 | | 1.0 | | 1.0 | |
| Change in % of device wear-time by sedentary time | 0.99 (0.96–1.02) | 0.641 | **0.95 (0.93**–**0.98)** | **0.001** | **1.05 (1.01–1.08)** | **0.005** | 1.04 (0.99–1.08) | 0.092 |
| Model statistics: |  | |  | |  | |  | |
| R² Nagelkerke | 0.347 | | 0.181 | | 0.113 | | 0.376 | |
| R² Cox&Snell | 0.239 | | 0.132 | | 0.076 | | 0.197 | |
| Hosmer&Lemeshow | 0.769 | | 0.663 | | 0.241 | | 0.624 | |

Note: Adjusted for the measurement season (age 15) and change in the device wear-time. Statistically significant odds ratios are in bold. Binary analysis: separately for each pattern (vs. all the others together). PA= physical activity.
