# Supplementary material for: Physical activity from adolescence to young adulthood: patterns of change, and their associations with activity domains and sedentary time
Source: Int J Behav Nutr Phys Act. 2021 Jun 30;18:85. doi: 10.1186/s12966-021-01130-x (PMC8246658; doi:10.1186/s12966-021-01130-x)
Supplement: Supplementary file 6 — Additional file 6. Associations between background variables and change in moderate-to-vigorous physical activity from age 15 to 19. [file 12966_2021_1130_MOESM6_ESM.docx]

**Additional file 6.**

|  | β Coefficient (95% CI) | *p* |
| --- | --- | --- |
| Living area at age 15: urban (reference: rural) | -0.85 (-9.0–7.3) | 0.837 |
| Self-rated health at age 19: good or excellent (reference: fair or poor) | **11.03 (0.5**–**21.5)** | **0.039** |
| Females (reference: males) | 3.37 (-5.1–11.8) | 0.431 |
| Change in sports club participation: neverᵃ (reference: maintenance/adopt) | -9.54 (-19.9–0.9) | 0.072 |
| Change in sports club participation: withdrawal (reference: maintenance/adopt) | **-12.00 (-21.2**– **-2.8)** | **0.011** |
| Change in active commuting: neverᵃ (reference: maintenance/adopt) | -7.47 (-17.1–2.2) | 0.127 |
| Change in active commuting: withdrawal (reference: maintenance/adopt) | -2.01 (-11.3–7.3) | 0.670 |
| Change in % of device wear-time by sedentary time | **-0.93 (-1.3– -0.6)** | **<0.001** |
| Adjusted R square = 0.402 | | |

Note: Linear regression analysis adjusted for change in the device wear-time, season at 1^st^ device-measurement, and baseline MVPA. Statistically significant odds ratios are in bold.

ᵃ *Never* indicates neither during the 1^st^ nor the 2^nd^ measurement.
